# Factors Affecting Family Planning Adoption in Migrant Communities: A Cross-Sectional Study From Central India

**DOI:** 10.7759/cureus.102445

**Published:** 2026-01-27

**Authors:** Sanjeev Kumar, Paharam Adhikari, Bikramjeet Mitra, Rajju Tiwari, Kalpana Arya, Ramlakhan Meena, Akanksha Karkhur

**Affiliations:** 1 Community Medicine, Government Medical College, Datia, Datia, IND; 2 Biochemistry, Government Medical College, Datia, Datia, IND

**Keywords:** community-based survey, health-seeking behavior, health service utilization, national family planning, reproductive health equity, unmet need for contraception, urban migrants

## Abstract

Background and objectives

Use of family planning methods among migrant communities is often low due to social disadvantage, frequent mobility, limited access to health services, and prevailing cultural beliefs. These factors contribute to a high unmet need for contraception and increase the risk of unintended pregnancies and related health problems. Evidence regarding knowledge, attitudes, practices, and barriers to family planning among migrant populations in Central India remains limited. Therefore, this study was undertaken to assess the knowledge, attitudes, and practices regarding family planning among the Lohpeeta migrant community in Datia, Central India, and to identify barriers influencing the adoption and use of family planning methods.

Methods

A descriptive cross-sectional study was conducted from December 1, 2023, to February 28, 2024, among all eligible individuals in the Lohpeeta community in India. Of 419 residents, 215 met the inclusion criteria. Data on knowledge, attitudes, and contraceptive practices were collected using a pre-tested questionnaire. Descriptive statistics were used to summarise the findings, and chi-square tests were used to assess associations, with p<0.05 considered significant.

Results

A total of 215 participants were included in the study, of whom 59.07% (n=127) were men and 40.93% (n=88) were women. The majority were married before the age of 18 years (n=175, 81.40%) and were illiterate (no formal education) (n=163, 75.81%). Awareness of contraceptives was higher among female participants (64.77%); however, overall current use of any contraceptive method was 27.44% (59/215), while regular use was much lower. Condoms (7.91%) and oral contraceptive pills (5.12%) were the most commonly used methods, while sterilisation was reported by 4.65% of the participants. A significant sex difference in attitudes was observed, with 47.24% of male versus 21.59% of female participants considering contraceptives useful (p<0.001).

Conclusion

Despite moderate awareness, contraceptive use in the community studied remains low due to low education, early marriage, and cultural norms. Policymakers should focus on culturally sensitive interventions, male involvement, mass media campaigns, and easy access to contraceptives to bridge the knowledge-practice gap in migrant and rural populations.

## Introduction

India is currently the most populous country in the world, having surpassed China in 2023 [1], and was the first country to initiate a national family planning programme in 1951-52 [2]. The programme, later renamed the National Family Planning Programme in 1952, aimed to reduce fertility to levels consistent with national economic development and population stabilisation goals [3].

Family planning remains central to reproductive health, maternal and child survival, gender equity, and sustainable socioeconomic development in India [4]. Despite decades of programmatic efforts, marked disparities persist in contraceptive awareness, access, and utilisation, particularly among socially marginalised groups such as migrant and tribal communities. Studies have highlighted that while awareness of modern contraceptive methods is generally high, a substantial gap exists between awareness, intention, and actual use [4,5]. Social norms, gendered decision-making, and misconceptions about side effects hinder uptake, even when services are available [6,7].

Evidence from tribal communities indicates lower contraceptive prevalence than the national average, with sterilisation remaining the dominant method of contraception [8]. Educational attainment, parity, access to media, and quality of family planning counselling strongly influence contraceptive choice and continuation [8,9]. High-quality counselling is associated with the greater adoption of effective methods, such as intrauterine devices and sterilisation, underscoring the importance of provider training [8].

Male negative attitudes toward contraception significantly reduce contraceptive use, often outweighing the effect of female empowerment in influencing family planning decisions [7]. Moreover, digital interventions such as Kilkari, a mobile-based messaging service implemented by ARMMAN in partnership with the Ministry of Health and Family Welfare, Government of India, have shown promise in increasing contraceptive awareness, but cultural barriers limit behavioural change [8,10]. Minimal knowledge of the ovulatory cycle and reproductive physiology remains a critical gap linked to limited education and health literacy among the tribal women [11].

Given the limited research on migrant populations, this study examines the knowledge, attitudes, and practices regarding family planning among the migrant and marginalised community in Datia, Madhya Pradesh, India, to inform culturally sensitive interventions for improving contraceptive uptake.

## Materials and methods

This was a descriptive cross-sectional study conducted from December 1, 2023, to February 28, 2024, among the Lohpeeta Rajasthani mobile tribe located in Datia, Madhya Pradesh, India. Ethical approval for the study was obtained before commencement from the Institutional Ethics Committee of Biomedical and Health Research in Human Participants, Government Medical College, Datia, Madhya Pradesh (approval number: 159/CM/GMC/IECBMHR/2023). The study was conducted in accordance with the ethical principles outlined in the Declaration of Helsinki. Participation was voluntary, and written informed consent was obtained from all eligible participants before enrolment. Participants were assured of confidentiality and anonymity, and no personal identifiers were recorded. Data were used solely for research purposes and were accessible only to the investigators.

Study population and sampling

The total population of the Lohpeeta migrant community residing in the study area was 419 individuals. A census-based approach was adopted, wherein all eligible individuals meeting the inclusion criteria were invited to participate, rather than selecting a sample. Individuals aged 18 years and above, who provided informed consent, were included.

Women who had attained menopause and men aged above 70 years were excluded. This exclusion was applied to focus the analysis on current contraceptive need, use, and continuation, which are most relevant within the biologically reproductive age group. While decision-making regarding family size may remain relevant beyond reproductive age, inclusion of individuals with no ongoing risk of conception could have led to misclassification of practice variables and dilution of estimates related to active family planning behaviour.

After applying these criteria, the final population comprised 215 individuals.

Study tool

Data were collected using a pre-tested, semi-structured, interviewer-administered questionnaire specifically developed for this study to assess factors influencing family planning adoption among migrant communities in Central India (see Appendices). The tool was designed to obtain comprehensive information on sociodemographic characteristics, migration-related factors, reproductive history, and knowledge, attitude, and practice related to family planning, along with accessibility of services, barriers to use, and decision-making dynamics.

Purpose of the Tool

The primary purpose of the questionnaire was to identify determinants associated with the adoption and non-adoption of family planning methods among migrant populations. It enabled systematic assessment of awareness, perceptions, practices, and contextual service-related and socio-cultural factors that may influence utilisation of family planning services.

Domains Covered

The questionnaire comprised 10 sections covering identification details, sociodemographic profile, migration profile, reproductive history, knowledge of family planning, attitude towards family planning, accessibility and service-related factors, practice of family planning, barriers and discontinuation, and decision-making and social support. The knowledge domain assessed awareness of family planning and familiarity with modern contraceptive methods and sources of information. The attitude domain explored perceptions regarding benefits, health concerns, religious beliefs, and shared decision-making in family planning. The practice domain captured current use, type of method, duration, and regularity of use. 

Method of Administration

Data were collected through face-to-face interviews using a semi-structured questionnaire. While interviewer-administered tools improve response completeness and comprehension in low-literacy populations, they may introduce social desirability bias, particularly for sensitive topics such as contraception and gendered attitudes.

To minimise this, investigators received focused training on neutral probing, non-judgmental interviewing, and maintaining privacy during interviews. Interviews were conducted in locations chosen by participants to ensure confidentiality. Female participants were interviewed in the absence of spouses or family members whenever feasible. These measures were intended to reduce response inhibition, especially in a patriarchal social setting where women may underreport negative attitudes or non-use of family planning.

Measurement and Scoring

Responses were analysed using a standardized knowledge-attitude-practice (KAP) framework. The knowledge domain assessed awareness of family planning, knowledge of modern contraceptive methods, and sources of information. Each correct or affirmative response was awarded one point, generating a knowledge score ranging from 0 to 8, categorised as poor (0-2), moderate (3-5), and good (6-8) knowledge.

The attitude domain comprised four Likert-scale items assessing perceived benefits of family planning, health concerns, religious beliefs, and shared decision-making. Responses were scored to maintain directional consistency, producing an attitude score ranging from 0 to 8, categorised as negative (0-3), neutral (4-6), and positive (7-8) attitude.

The practice domain evaluated the current use of family planning, the type of method used, the duration of use, and the consistency of use. Greater weight was assigned to current contraceptive use to reflect its public health relevance, resulting in a practice score ranging from 0 to 5, categorised as poor (0-1), fair (2-3), and good (4-5) practice.

Validity and Reliability

Content validity of the questionnaire was assessed through expert review by three faculty members from the Department of Community Medicine, Government Medical College, Datia, who independently evaluated each item for relevance, clarity, cultural appropriateness, and alignment with study objectives. Based on their feedback, minor modifications were made to item wording and sequencing to improve comprehension and contextual relevance.

Face validity and feasibility were further examined through pilot testing conducted among 20 individuals from a sociodemographically comparable migrant population not included in the final study. The pilot resulted in refinement of ambiguous terms, simplification of response options, and reordering of selected questions to improve interview flow. Given the exploratory nature of the study and the formative construction of the tool, formal quantitative indices such as the Content Validity Index were not calculated.

Reliability was assessed using an approach appropriate to the binary and formative nature of the questionnaire. For the knowledge domain, which consisted predominantly of dichotomous (Yes/No) items, internal consistency was evaluated conceptually using the Kuder-Richardson Formula 20 (KR-20), which is more suitable than Cronbach’s alpha for binary responses [12]. However, as the knowledge and practice domains were designed as formative indices capturing multiple independent aspects of family planning awareness and behaviour, high inter-item correlation was neither expected nor required.

Accordingly, reliability was primarily ensured through clear item construction, expert validation, pilot testing, and standardised interviewer training, consistent with approaches used in large-scale surveys such as the National Family Health Survey.

Data entry and statistical analysis

The data were compiled and entered into Microsoft Excel (Microsoft Corporation, Redmond, Washington, United States), and then imported into the statistical software Jamovi version 2.3.28 (The Jamovi project, Sydney, Australia) for analysis. Descriptive statistics, such as frequencies and percentages, were used to summarise the participants’socio-demographic details and contraceptive knowledge, attitudes, and practices. Chi-square test was used to assess the associations between categorical variables. Statistical significance was set at p < 0.05.

## Results

Of the 215 participants, 127 were men (59.07%) and 88 were women (40.93%). The mean age of the participants was 35.9 ± 10.6 years. Most participants were married (97.67%), while five (2.33%) were unmarried. Among married participants, 175 (83.33%) were married before the age of 18 years. With respect to duration of marriage, 58 (27.62%) had been married for less than two years, 108 (51.43%) for two to five years, and 44 (20.95%) for more than five years. Regarding educational status, 163 participants (77.62%) had no formal education, 44 (20.95%) had completed primary education, and eight (3.81%) had completed secondary education (Table 1).

**Table 1:** Distribution of participants according to socio-demographic characteristics (N=215) The values are expressed as frequency (n) and percentage (%), except for age, which is presented as mean ± standard deviation. † Age at marriage and duration of marriage were assessed only among currently married participants (n = 210). Percentages for these variables are calculated using n = 210 as the denominator.

Variable	Frequency	Percentage
Gender		
Male	127	59.07%
Female	88	40.93%
Age (years), mean ± SD	35.9 ± 10.6	—
Age group		
18–30	80	37.21%
31–42	71	33.02%
>42	64	29.77%
Marital status		
Married	210	97.67%
Unmarried	5	2.33%
Age at marriage (n=210)^†^		
<18 years	175	83.33%
>18 years	35	16.67%
Years of marriage (n=210)^†^		
<2 years	58	27.62%
2–5 years	108	51.43%
>5 years	44	20.95%
Education		
Illiterate (no formal education)	163	75.81%
Primary	44	20.47%
Secondary	8	3.72%

Awareness of contraceptives was higher among women (64.77%) compared to men (55.12%) (p=0.186). Both groups generally exhibited low knowledge of certain methods and types of contraceptives. In terms of attitudes, a significant difference emerged between male and female participants (p<0.001), with 47.24% of male participants viewing contraceptives as useful, in contrast to 21.59% of female participants. Regarding practice, the majority reported never using contraceptives (70.08% of men and 76.14% of women; p=0.141) (Table 2).

**Table 2:** Distribution of population according to knowledge, attitude and practice of contraceptive (N=215) Chi-square test was used to test the association NS: not significant (p>0.05); *** p<0.001 (significant)

Variable	Male (n=127), n (%)	Female (n=88), n (%)	X^2 #^	p-value
Knowledge about contraceptive				
Heard about contraceptive	70 (55.12%)	57 (64.77%)	3.359	0.186 ^NS^
Know some methods of contraceptive	32 (25.19%)	22 (25%)		
Know about type of contraceptive	24 (18.89%)	9 (10.23%)		
Attitude toward contraceptive				
It is useful	60 (47.24%)	19 (21.59%)	73.76	<0.001***
It is not a good thing	13 (10.24%)	58 (65.90%)		
Responsibility of both partners	17 (13.39%)	4 (4.55%)		
Only males need to use it	8 (6.30%)	1 (1.14%)		
Only females need to use it	29 (22.83%)	6 (6.82%)		
Practice				
Never use	89 (70.08%)	67 (76.14%)	6.895	0.141 ^NS^
Use during first contact	11 (8.66)	8 (9.09%)		
Use during the last contact	8 (6.30%)	0 (0)		
Regular use	6 (4.72%)	2 (2.27%)		
Use occasionally	13 (10.24%)	11 (12.5%)		

**Figure 1:**
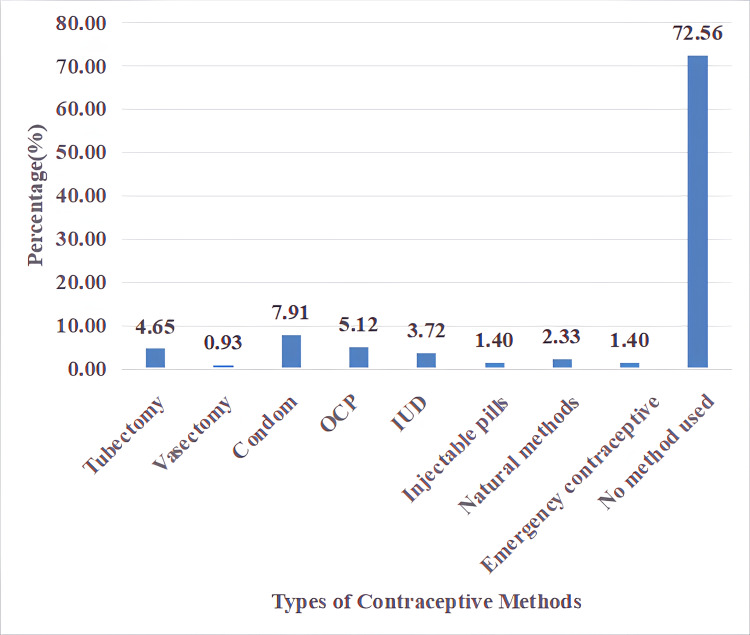
Type of contraceptive methods used Data shown as percentages OCP: oral contraceptive pills; IUD: intrauterine device

With regard to contraceptive methods, most reported using condoms (7.91%, n = 17). Next were birth control pills (5.12%, n = 11), tubectomy (4.65%, n = 10), and IUDs (3.72%, n = 8). Natural methods were used by 2.33% (n = 5), and injectable pills and emergency contraceptives were used by 1.40% (n = 3) each. A large number of participants (72.56%, n = 156) did not use any birth control method (Figure 1).

## Discussion

The study of the Lohpeeta migrant community in Datia revealed a significant gap between awareness of contraception and its actual use. While condoms and birth control pills were frequently mentioned, their usage was limited. According to the National Family Health Survey, Round 5 (NFHS-5) data (2019-21), 78.8% of tribal women lacked knowledge about the ovulatory cycle. Factors such as higher education, a partner’s education, media exposure, and current contraceptive use were associated with improved reproductive knowledge [13]. NFHS-4 (2015-16) data indicated that 86.5% of women in India had their birth control needs met. Younger, better-educated women tended to prefer modern reversible methods, although this preference varied by region [14]. In Madhya Pradesh, NFHS-5 reported a contraceptive use rate of about 72% among married women, significantly higher than that of the Lohpeeta community [15]. This highlights ongoing disparities in family planning among marginalised migrant groups. Additionally, mass media exposure significantly increased awareness and use of contraceptives in India [16].

In this study, a significant number of participants (81.40%) reported marrying before the age of 18, highlighting a critical issue since child marriage is illegal in India. Early marriage can disrupt education, diminish independence, lead to early pregnancies, and restrict women's choices. These challenges are exacerbated in migrant and impoverished areas where access to education and health services is limited, and gender roles heavily influence reproductive decisions.

The study also revealed that a substantial portion of participants (75.81%) were illiterate, further compounding the issues associated with early marriage. Merely knowing about contraception is insufficient without robust social and economic support. Sharma et al. identified low education, poverty, and inadequate health services as major contributors to unmet family planning needs among young married women in India [17]. There are distinct gender disparities in attitudes toward contraception; nearly half of the men (47.24%) viewed contraceptives as beneficial, whereas only 21.59% of women shared this sentiment (p<0.001). This contrasts with previous findings where female sterilisation was prevalent, particularly among less educated women with larger families [18].

In this study, only 4.65% underwent tubectomy, indicating a gradual shift toward reversible methods, though usage remains low due to social norms, misconceptions, and limited female empowerment. National studies indicate a decline in unmet family planning needs [19], with an increase in the use of reversible contraceptives among younger, educated, and wealthier women [20]. However, contraceptive use in this study group (27.44%) is significantly lower than NFHS-5 estimates, underscoring that migrant and impoverished communities are still excluded from national family planning benefits [13]. Other studies also highlight that family pressure, particularly from in-laws, and cultural expectations significantly influence fertility behaviour in India [14].

In this group, early marriage may further clarify the low use of contraceptives. Longitudinal studies indicate that young married women who plan to postpone or limit having children are more inclined to start using contraception over time, while an unmet need alone does not consistently lead to its adoption. Women who are pregnant or already have at least one child at the beginning are more likely to begin family planning later [20]. These results highlight the importance of early, context-specific strategies that tackle structural, cultural, and gender-related obstacles to contraceptive use in migrant communities.

Limitations

When examining the results of this study, it is important to consider potential biases. Due to the cross-sectional nature of the study, we cannot definitively establish causal relationships between knowledge, attitudes, and contraceptive practices. The data, derived from self-reports, may lack accuracy, as participants might forget details or provide responses they believe are socially acceptable. This could lead to underreporting of contraceptive use or overreporting of knowledge. Discussing sensitive topics like family planning in person might prompt individuals, particularly women in male-dominated societies, to offer responses they perceive as expected, despite our efforts to ensure privacy and train interviewers to mitigate this effect. The study predominantly involved married individuals (97.67%), which may limit the applicability of the findings to unmarried individuals with different contraceptive needs. Additionally, we were unable to fully account for factors such as education, age at marriage, number of children, access to health services, and cultural norms, all of which could influence the results. The differing perspectives of men and women on contraception might stem from restricted female autonomy, early marriages, and male-dominated decision-making within the study group.

## Conclusions

Although awareness of family planning exists within the Lohpeeta tribal community of Datia, contraceptive use remains limited due to low educational attainment, early marriage, and socio-cultural beliefs. To address these gaps, strengthened public-private sector partnerships are needed to implement tailored community outreach programmes that specifically target migrant populations and address high levels of illiteracy. These efforts should be supported by the active involvement of men in family planning decision-making and complemented by culturally sensitive public health communication campaigns, delivered through locally appropriate media and community leaders, to dispel misconceptions and improve access to contraceptive services.

## Appendices

Study questionnaire 

**Table 3:** Semi-Structured Questionnaire for Assessment of Factors Affecting Family Planning Adoption among Migrant Communities in Central India ☐ indicates multiple-choice responses to be ticked by the participant. MCQ items allow only one most appropriate response unless specified as “Multiple answers allowed.” Likert-scale items under Section F were used to assess attitude, with responses categorised as Agree, Neutral, or Disagree. Scoring Method Knowledge Score (0–8): Derived from items E1–E4. One point was awarded for each correct response to E1 and E2, one point for each correctly identified modern family planning method in E3 (maximum 5 points), and one point for a health worker–based source of information in E4 (ASHA/health staff). Scores were categorized as Poor (0–2), Moderate (3–5), and Good (6–8). Attitude Score (0–8): Derived from items F1–F4 using a 3-point Likert scale. Positive statements (F1, F4) were scored as Agree = 2, Neutral = 1, Disagree = 0. Negative statements (F2, F3) were reverse-scored. Scores were categorized as Negative (0–3), Neutral (4–6), and Positive (7–8). Practice Score (0–5): Derived from items H1–H4. Current use of any FP method (H1) was scored as Yes = 2, No = 0; use of a modern FP method (H2) = 1; duration of use ≥6 months (H3) = 1; and regular use (H4) = 1. Scores were categorized as Poor (0–1), Fair (2–3), and Good (4–5). FP: family planning; ASHA: Accredited Social Health Activist

Section	Item	Question / Statement	Response Format
SECTION A: Identification	A1	Participant ID	__________
	A2	Date of Interview	___ / ___ / ____
	A3	Place of Interview	☐ Home ☐ Worksite ☐ Camp
	A4	Type of Migration	☐ Seasonal ☐ Circular ☐ Permanent
SECTION B: Socio-Demographic Profile	B1	Age (in years)	______
	B2	Sex	☐ Male ☐ Female
	B3	Marital status	☐ Married ☐ Unmarried
	B4	Religion	☐ Hindu ☐ Muslim ☐ Others
	B5	Education	☐ Illiterate ☐ Primary ☐ Secondary ☐ Higher
	B6	Occupation	☐ Construction ☐ Factory ☐ Agricultural ☐ Others
	B7	Monthly family income (₹)	☐ <10,000 ☐ 10,000–20,000 ☐ >20,000
SECTION C: Migration Profile	C1	Place of origin	____________________
	C2	Duration of stay at current location	☐ <6 months ☐ 6–12 months ☐ >1 year
	C3	Reason for migration	☐ Employment ☐ Family ☐ Others
	C4	Is there any health facility near your worksite?	☐ Yes ☐ No
SECTION D: Reproductive History	D1	Age at marriage	______ years
	D2	Number of living children	______
	D3	Desired family size	☐ 1 ☐ 2 ☐ ≥3
	D4	Do you desire more children?	☐ Yes ☐ No
SECTION E: Knowledge of Family Planning	E1	Have you heard about family planning?	☐ Yes ☐ No
	E2	Do you know at least one modern FP method?	☐ Yes ☐ No
	E3	Which FP methods do you know? (Multiple answers allowed)	☐ Condom ☐ Oral pills ☐ IUCD ☐ Injectable ☐ Sterilization
	E4	Source of information (tick main source)	☐ ASHA ☐ Health staff ☐ Media ☐ Friends/Relatives
SECTION F: Attitude towards Family Planning	F1	FP is beneficial for family health	☐ Agree ☐ Neutral ☐ Disagree
	F2	FP causes health problems	☐ Agree ☐ Neutral ☐ Disagree
	F3	FP is against religious beliefs	☐ Agree ☐ Neutral ☐ Disagree
	F4	Both partners should decide FP use	☐ Agree ☐ Neutral ☐ Disagree
SECTION G: Accessibility & Service Factors	G1	Are FP methods available near your residence/worksite?	☐ Yes ☐ No
	G2	Distance to nearest FP service	☐ <2 km ☐ 2–5 km ☐ >5 km
	G3	Did ASHA visit you in last 6 months?	☐ Yes ☐ No
	G4	Have you received FP counselling?	☐ Yes ☐ No
SECTION H: Practice of Family Planning	H1	Are you currently using any FP method?	☐ Yes ☐ No
	H2	If yes, which method?	☐ Condom ☐ Oral pills ☐ IUCD ☐ Injectable ☐ Sterilization
	H3	Duration of use	☐ <6 months ☐ ≥6 months
	H4	Consistency of use	☐ Regular ☐ Irregular
SECTION I: Barriers and Discontinuation	I1	If not using FP, what is the main reason?	☐ Desire for child ☐ Fear of side effects ☐ Partner refusal ☐ Religious belief ☐ Non-availability
	I2	Have you experienced any side effects?	☐ Yes ☐ No
	I3	Have you discontinued any FP method previously?	☐ Yes ☐ No
SECTION J: Decision-Making & Social Support	J1	Who mainly decides FP use in your family?	☐ Self ☐ Spouse ☐ Joint ☐ Family elders
	J2	Do you receive spousal support for FP use?	☐ Yes ☐ No
	J3	Do peers influence your FP decision?	☐ Yes ☐ No
